# Comparison of *Streptococcus halichoeri* isolates from canine and fur animal infections: biochemical patterns, molecular characteristics and genetic relatedness

**DOI:** 10.1186/s13028-020-00525-3

**Published:** 2020-06-03

**Authors:** Marjut Eklund, Kirsi Aaltonen, Tarja Sironen, Mirja Raunio-Saarnisto, Thomas Grönthal, Heli Nordgren, Anna Pitkälä, Olli Vapalahti, Merja Rantala

**Affiliations:** 1 Department of Equine and Small Animal Medicine, Faculty of Veterinary Medicine, University of Helsinki, P.O. Box 57, 00014 Helsinki, Finland; 2 Department of Virology, Faculty of Medicine, University of Helsinki, P.O. Box 21, 00014 Helsinki, Finland; 3 Department of Veterinary Biosciences, Faculty of Veterinary Medicine, University of Helsinki, P.O. Box 66, 00014 Helsinki, Finland; 4 Veterinary Bacteriology and Pathology Research Unit, Finnish Food Authority, P.O. Box 100, 60100 Seinäjoki, Finland; 5 HUSLAB, Hospital District of Helsinki and Uusimaa, P.O. Box 100, 00029 Helsinki, Finland

**Keywords:** Bacterial typing, Blue fox, Dog, Finnraccoon, Mink, Streptococcus

## Abstract

**Background:**

*Streptococcus halichoeri* infections have been reported in grey seals, a European badger, a Stellar sea lion and humans, but its presence in companion and fur animals is unknown. Since 2010, *S. halichoeri*-like bacteria (SHL) have been isolated from fur animals and dogs in Finland. Our aim was to retrospectively investigate laboratory records for SHL from canine and fur animal infections, characterize the isolates and compare their genetic relatedness in relation to three reference strains: CCUG 48324^T^, originating from a grey seal, and strains 67100 and 61265, originally isolated from humans.

**Results:**

A total of 138 and 36 SHLs from canine and fur animal infections, respectively, were identified in the laboratory records. SHL was commonly associated with skin infections, but rarely as the only species. A set of 49 canine and 23 fur animal SHLs were further characterized. MALDI-TOF confirmed them as being *S. halichoeri*. The growth characteristics were consistent with the original findings, but isolates were catalase positive. In total, 17 distinct API 20 Strep patterns were recorded among all 75 isolates tested, of which pattern 5563100 was the most common (n = 30). Antimicrobial resistance to erythromycin and clindamycin was common in canine isolates, but rare in fur animal isolates. Three clusters were observed by PFGE, and 16S rRNA sequencing revealed 98.1–100% similarities with the human strains and 98.1–99.5% with the seal strain. A phylogenetic tree of concatenated 16S rRNA and *rpoB* revealed closely related isolates with two clades. Fifteen canine isolates were identical to the human strains based on concatenated 16S rRNA and *rpoB* sequencing.

**Conclusions:**

*Streptococcus halichoeri* appears to be quite a common bacterial species in the skin of dogs and fur animals. The clinical significance of *S. halichoeri* is uncertain, as it was rarely isolated as a monoculture. No apparent temporal or spatial clustering was detected, but isolates from different sources were genetically very similar. Because many canine isolates were genetically similar to the human reference strains, transmission between dogs and humans may be possible. WGS sequencing of strains from different sources is needed to further investigate the epidemiology and virulence of *S. halichoeri.*

## Background

*Streptococcus halichoeri* is a Lancefield Group B streptococcus that was first described in 2004 from post-mortem and clinical specimens of grey seals (*Halichoerus grypus*) [1]. Since then, it has been isolated from wild animals, including a European badger (*Meles meles*) with pyogranulomatous pleuropneumonia [2], and from the kidney of a dead Steller sea lion (*Eumetopias jubatus*) [3]. *S. halichoeri* has also been associated with severe human infections such as postoperative empyema [4], and from blood, wound and sinus infections [5], vulvar abscess and paronychia [6] and infectious cellulitis [7]. Due to pheno- and genotypic differences between strains of animal and human origin, Shewmaker et al. [5] suggested that this bacterial species should be divided into two subspecies: *S. halichoeri* subsp. *halichoeri* and *S. halichoeri* “subsp. *hominis*”. To our knowledge, there are no reports of *S. halichoeri* infections in domesticated animals.

In 2010, the Finnish Food Authority’s laboratory isolated three Gram-positive cocci similar, but not identical to *S. halichoeri* (98.6% similarity to *S. halichoeri* by 16S rRNA sequencing) simultaneously with *Arcanobacterium phocae* in specimens from fur animal epidemic necrotic pyoderma (FENP) [8]. In December 2010, the first *S. halichoeri*-like bacterium (SHL) from a canine ear swab specimen taken due to otitis externa (M. Rantala, personal communication) was identified based on 99.5% 16S rRNA gene sequence similarity (525 base pairs of the V1–V3 region) to the *S. halichoeri* type strain CCUG 48324^T^ originating from a grey seal. In contrast to the type strain, a positive catalase reaction was observed. Since then, additional SHL isolates have been identified at the clinical microbiology laboratory (CML) of the Faculty of Veterinary Medicine, University of Helsinki, and at the Finnish Food Authority from several canine and fur animal clinical specimens. The purpose of this study was to retrospectively investigate the number of SHL findings in laboratory records, characterize SHL isolates with biochemical and molecular methods and examine their genetic relatedness.

## Methods

### *Frequency and description of* Streptococcus halichoeri-*like findings and literature search*

A database search was performed from the laboratory information systems of the CML and the Finnish Food Authority covering the period from 1 January 2010 to 31 December 2016 to identify laboratory reports in which SHL bacteria had been recorded. Due to a database update at the CML in June 2011, some earlier isolates may have been overlooked. The search included records of “Group B streptococcus” or “*Streptococcus* sp.” with an annotation of the SHL bacterium. In addition, information on the animal species, gender, specimen type, sampling site and number of bacterial species per report, as well as disk diffusion susceptibility testing results, was collected from laboratory records. The diffusion susceptibility testing had been performed according to CLSI standards [9] (interpreted by using the susceptibility breakpoints for human beta-haemolytic streptococci [10]) for clindamycin (CLI ≥ 19 mm), erythromycin (ERY ≥ 21 mm), penicillin G (PEN ≥ 24 mm), tetracycline (TET ≥ 23 mm) and trimethoprim–sulfamethoxazole (SXT ≥ 16 mm). Information on gender and susceptibility testing results were not available for fur animal SHL reports. Prior publications regarding *S. halichoeri* were obtained by using PubMed. In addition, a Google search was conducted to find those publications not indexed in PubMed using the search words “*Streptococcus halichoeri*”.

The clinical isolates had been presumptively identified as SHL bacteria if the colony morphology was consistent with the description of *S. halichoeri* subsp. *halichoeri* [1], i.e. white to greyish white, umbonate, non-haemolytic and with a colony diameter of 0.5 mm after 24 h of incubation on tryptic soy agar (TSA) with 5% sheep blood (Oxoid Ltd, Basingstoke, UK), and agglutination with the Group B Lancefield antisera (Streptococcal Grouping Kit, Oxoid Ltd). In addition, a positive Christie-Atkin-Munch-Petersen (CAMP) reaction against *Staphylococcus aureus* ATCC^®^ 25923 was used for preliminary identification. In contrast to the species description by Lawson et al. [1], positive catalase reactions with 3% H_2_O_2_ on glass slides after growing on a blood-agar base had been recorded.

### Bacterial strains for biochemical and molecular characterizations

Out of a total of 138 CML reports in which SHL that had been recorded, 49 clinical isolates of canine origin had been stored in skim milk at − 80 °C in the laboratory’s culture collection and were available for biochemical and molecular analyses. Fur animal SHL isolates (n = 23) were from minks (*Neovison vison*, n = 12), blue foxes (*Vulpes lagopus*, n = 6) and Finnraccoons (*Nyctereutes procyonoides*, n = 5). *S. halichoeri* subsp. *halichoeri* CCUG 48324^T^ isolated from a grey seal (*Halichoerus grypus*) [1], *S. halichoeri* “subsp. *hominis*” CCUG 67100 isolated from a human in North Carolina, USA, and another human isolate, CCUG 61265, from Kalmar in Sweden [5] were used as reference strains. Fresh overnight cultures on 5% TSA sheep blood agar (Oxoid Ltd) were used for biochemical and molecular characterizations.

### Biochemical characterization and identification with MALDI-TOF

Biochemical characterization was performed with API 20 Strep (bioMérieux, Marcy l’Etoile, France) as instructed by the manufacturer, and the obtained profiles were assessed with APIWEB™ software version 1.4.0 (bioMérieux, SA). Catalase activity was tested with 3% H_2_O_2_ on glass slides after culturing an isolate on a 5% TSA sheep blood agar and a non-blood agar base (UriSelect4, Bio-Rad, France). The isolates were identified by using a matrix-assisted laser desorption/ionization time-of-flight mass spectrometry (MALDI-TOF) device (Bruker MALDI Biotyper Microflex LT, Bruker Daltonik GmbH, Bremen, Germany). MALDI Biotype MSP Identification Standard Method v 1.1 was followed to prepare the samples on MBT Biotarget 96 plates (Bruker Daltonik GmbH). Bruker Matrix HCCA (IVD) was used as the matrix. The Bruker Bacterial Test Standard (IVD) (Bruker Daltonik GmbH) was used for instrument calibration. Mass spectra were analysed in a mass/charge range from 2000 to 20,000 Da with MBT Compass v4.1 on flexControl v3.4 (Bruker Daltonik GmbH) using BDAL-7311 as the reference library. MALDI-TOF score values ≥ 2.000 were used for species-level identification. Susceptibility testing was performed for fur animal isolates as described above.

### Molecular characterizations

#### Pulsed-field gel electrophoresis (PFGE)

*SmaI* (New England Biolabs Inc., MA, USA) restriction enzyme patterns of the clinical isolates and reference strains were investigated by PFGE according to a published protocol for *Streptococcus pneumoniae* [11], with minor modifications. Bacterial suspensions of 8.0–9.0 McFarland density were embedded in SeaKem Gold (Lonza, Rockland, ME, USA) agarose. The electrophoresis run time was 16 h. SYBR Safe (Invitrogen, CA, USA) was used to stain the DNA fragments and GelCompar II software (version 6.6 Applied Maths NV, Belgium) was used to examine the PFGE patterns. Analysis was conducted using the unweighted pair grouping method with arithmetic mean (UPGMA) using the Dice similarity coefficient, and optimization and position tolerance were both set at 1.5%. Clonal clusters were determined by using an 85% similarity cut-off [12]. ATCC 49619 *Streptococcus pneumoniae* was used to root a phylogenetic tree. Based on the PFGE analysis, representative isolates from each cluster, including their sub-clusters, were selected for further sequence analysis of 16S rRNA and *rpoB* genes.

### PCR and sequencing of 16S rRNA and rpoB genes

The DNA isolation, amplification and sequencing of the nearly complete 16S rRNA gene (nucleotides 8–1541, ca. 1500 base pairs, bp) was performed as described previously [13]. For amplification of an approximately 1200 bp fragment of the *rpoB* gene, the PCR reaction mix contained 1.5 µL of DNA template, 10 µL of Phusion Flash High Fidelity Master Mix (Thermo Fisher Scientific, Waltham, Massachusetts, USA), 0.38 µM of forward 5ʹ-ATGGGTGCCAACATGCA-3ʹ and reverse 5ʹ-GCCCAAACTTCCATCTC-3ʹ primers (Shewmaker P. and Whitney A., personal communication) based on the published *rpoB* genome of *S. halichoeri* accession number KP890283 [5]. PCR conditions were similar to those for 16S rRNA [13], except for annealing at 54 °C (10 s) and elongation at 72 °C (25 s). The *rpoB* gene of three isolates could not be amplified with this protocol, and a primer pair of UnivrpoB3F 5ʹ-ATGGGNDCNAAYATGCA-3ʹ and UnivrpoB23R 5ʹ-GCYCANVHYTCCATYTC-3ʹ [14] was therefore used for these isolates. This PCR reaction mix contained 5 µL of DNA template, 25 µL of DreamTaq Master Mix (2X) (Thermo Fisher Scientific) and 1 µM of primers with the following PCR conditions: 95 °C (3 min); 40 cycles of 95 °C (30 s), 45 °C (30 s) and 72 °C (1 min); and final elongation at 72 °C (15 min). The PCR products were purified with ExoI and FastAP protocol according to the manufacturer’s instructions (Thermo Fisher Scientific), after which sequencing primers were added. Sequencing of *rpoB* was carried out with the same primers as the respective PCR reactions. Sequencing was performed at a commercial laboratory (Macrogen Inc., Amsterdam, Netherlands) with an ABI 3730 XL automated sequencer. Sequences of CCUG reference strains were derived from NCBI GenBank (Table 1). The sequences were analysed with CLC Main Workbench software (version 7.5.1, Qiagen, Copenhagen, Denmark) and trimmed (16S rRNA 1400 bp, starting from 61 bp, and *rpoB* 1039 bp, starting from 62 bp) for further analysis. Trimmed 16S rRNA sequences were compared with the NCBI GenBank database for bacterial identification.

**Table 1:** Reference 16S rRNA and *rpoB* gene sequences from the National Center for Biotechnology Information (NCBI) database

Strain	NCBI sequence accession numbers (URL)		References
	16S rRNA	*rpoB*	
*S. halichoeri* subsp. *halichoeri* CCUG 48324^T^	AJ606046 (https://www.ncbi.nlm.nih.gov/nuccore/AJ606046)	KP890283 (https://www.ncbi.nlm.nih.gov/nuccore/KP890283)	[1] (16S rRNA), [5] (*rpoB*)
*S. halichoeri* “subsp. *hominis*” CCUG 67100 (SS1844)	KP851845 (https://www.ncbi.nlm.nih.gov/nuccore/KP851845)	KP890278 (https://www.ncbi.nlm.nih.gov/nuccore/KP890278)	[5]
*S. halichoeri* “subsp. *hominis*” CCUG 61265	KP851848 (https://www.ncbi.nlm.nih.gov/nuccore/KP851848)	KP890282 (https://www.ncbi.nlm.nih.gov/nuccore/KP890282)	[5]
*S. agalactiae* CU_GBS_08	NZ_CP010874 (https://www.ncbi.nlm.nih.gov/nuccore/NZ_CP010874)	CP010874 (https://www.ncbi.nlm.nih.gov/nuccore/CP010874)	None
*S. canis* FSLZ3-227	NZ_AIDX01000001.2 (https://www.ncbi.nlm.nih.gov/nuccore/NZ_AIDX01000001.2)	NZ_AIDX01000001 (https://www.ncbi.nlm.nih.gov/nuccore/NZ_AIDX01000001)	[21]

## 16S rRNA and rpoB phylogeny

To examine the phylogenetic relationship of the isolates, the trimmed 16S rRNA and *rpoB* sequences of 38 isolates representing different PFGE patterns were combined, and the concatenated sequences (2439 bp in length) were subjected to multiple sequence alignment using Clustal X in Mega 7.0. A phylogenetic tree was built using the maximum likelihood approach in Mega with 1000 bootstraps. The respective sequences of the *S. halichoeri* reference strains (CCUG 48324^T^, CCUG 61265 and CCUG 67100), *Streptococcus canis* (FSLZ3-227) and *Streptococcus agalactiae* (CU_GBS_08) published in the GenBank database were included in the analysis (Table 1).

## Results

During the defined period, 138 SHL findings were recorded among 22,757 laboratory reports (out of which 16 158 [70%] were from dogs) at the CML. All the SHL findings were from specimens taken from dogs, 64% of which were female. The majority of specimens with an SHL finding (85%) were from the Helsinki Metropolian Area. Of these, 68% were from private clinics, 17% from the Veterinary Teaching Hospital of the University of Helsinki, and the rest (15%) were from private clinics situated all over Finland. In addition to SHL, all 138 specimens contained polymicrobial growth with one to five other bacterial species, most commonly with *Staphylococcus pseudintermedius*. The sources for the 138 SHL-containing specimens were superficial pus (n = 121; 88%), deep pus specimens (n = 15; 11%) and other specimens (n = 2; 1%). Of the specific infection sites, the most frequent was the ear canal (n = 62; 45%), followed by various skin lesions (n = 42; 30%; furuncles, skin abrasions, abscesses, wounds). The rest (n = 34, 25%) were from various infection sites or the site was not reported. Susceptibility results were available for 136 canine isolates for all antimicrobials, except for tetracycline, for which only 71 isolates had been tested (tetracycline testing initiated in 2015). All isolates were susceptible to PEN and SXT. Seventy-five isolates out of 136 (55%) expressed resistance to both ERY and CLI, suggesting a common resistance mechanism. A histogram of the plotted tetracycline results displayed a bi-modal distribution in which zone sizes for the susceptible population ranged from 25 to 40 mm (n = 28/71; 39%) and those for the non-susceptible population from 6 to 15 mm (n = 43/71; 61%). Thirty-seven TET R isolates also showed resistance to ERY and CLI.

Regarding fur animals, 547 shipments consisting of fur animal necropsy or organ samples were investigated at the Food Authority between June 2011 and December 2016. SHL findings were recorded in 36 animals from 26 farms. The vast majority of the farms (n = 22) were located in the Ostrobothnia area, where the Finnish fur animal industry is concentrated. Of the 36 animals, 28 were minks and eight were blue foxes. Eleven minks had FENP, eight expressed other skin infections, five had sepsis, two had pyothorax, one had trauma with inflammation and in one case the diagnosis was not available. In the majority of mink cases, SHL was isolated together with other bacteria (with *Arcanobacterium phocae*, n = 18; in mixed growth, n = 5; and with *Streptococcus canis*, n = 1), while only four specimens yielded SHL as a pure or almost pure culture. Specimens from blue foxes were associated with FENP (n = 4) and other miscellaneous conditions (n = 3; pneumonia, metritis and conjunctivitis, skin infection with conjunctivitis, and in one case the data were missing). In these specimens, SHL was frequently associated with other bacteria (*A. phocae*, n = 5; *S. canis*, n = 1; multiple species, n = 1; pure growth, n = 1). No SHL findings from Finnraccoons were made after 2010 and early 2011 from FENP specimens taken from this species. Gross pathology findings from fur animals in which *S. halichoeri* was isolated as a pure or almost pure growth are listed in Table 2.

**Table 2:** Gross pathology findings from fur animals in which *Streptococcus halichoeri* was isolated as a pure or almost pure growth

Strain	Species	Organ	Clinical presentation	Pathological findings
Strep B-17	Mink	Lung	Found dead	Pneumonia, enteritis, multiple abscesses in liver and spleen, splenomegaly
Strep B-18	Mink	Spleen	Found dead	Pyothorax, pneumonia, enteritis
Strep B-19	Mink	Lung	Found dead	Pleuritis, pneumonia
Strep B-21	Blue fox	Skin	N/A	Dermatitis

*N/A* not available

**Fig. 1:**
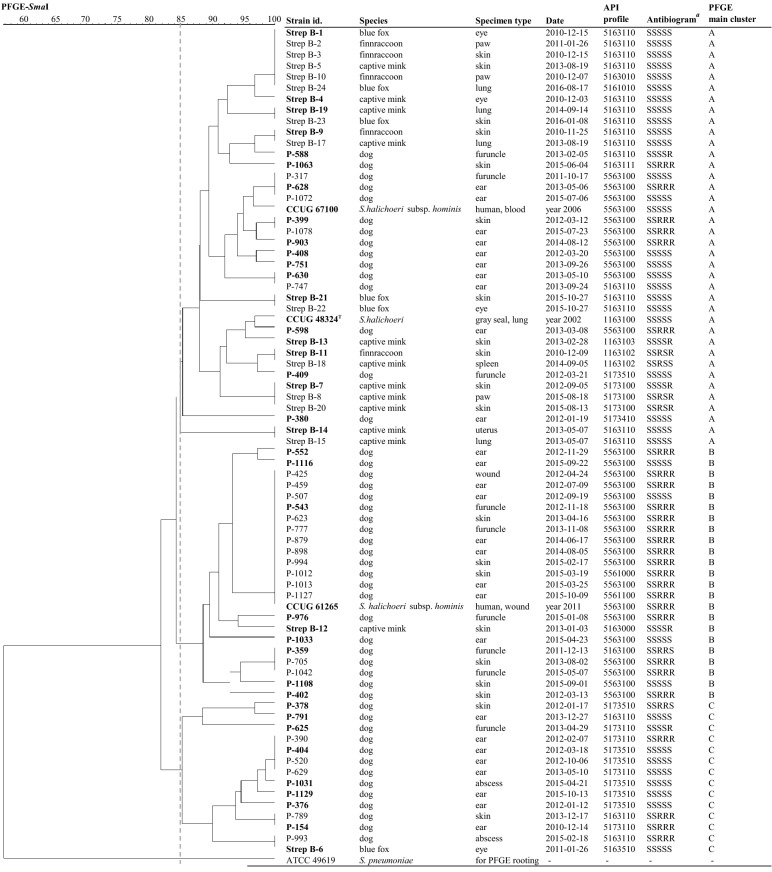
PFGE clustering, API 20 Strep and antimicrobial susceptibility profiles of the *S. halichoeri* strains studied. Clonal clusters were determined by using an 85% similarity cut-off, which is marked as a dashed vertical line. Isolates designated with a bold font were selected for the phylogenetic analysis of the 16S rRNA and *rpoB* sequences (Fig. 2). ^a^The antibiogram is presented in the following order: benzylpenicillin, trimethoprim-sulfamethoxazole, erythromycin, clindamycin and tetracycline

**Fig. 2:**
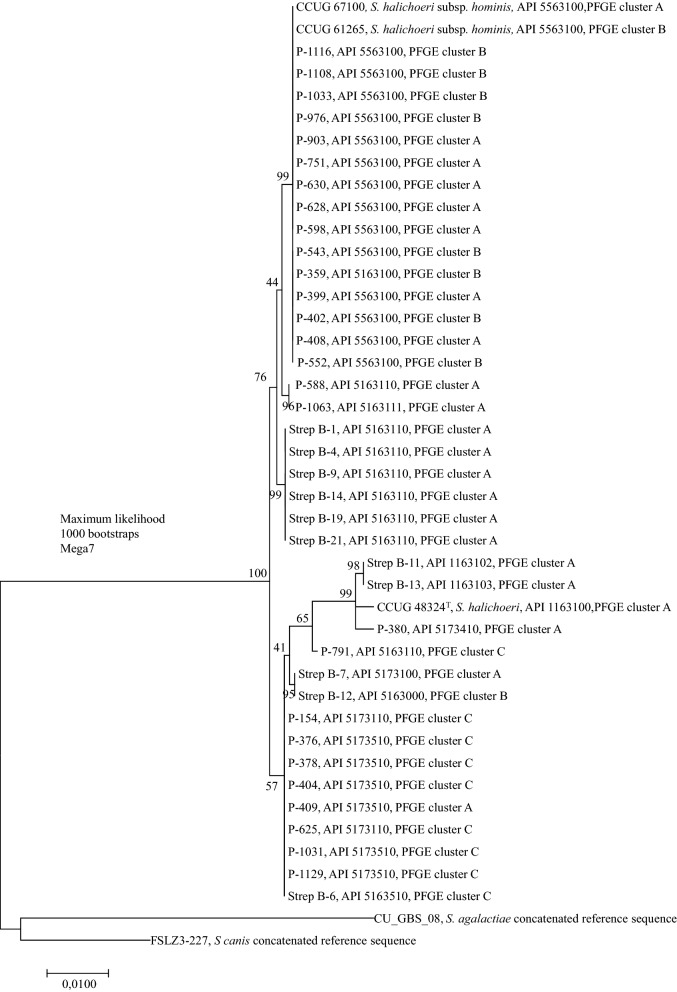
Phylogenetic tree of concatenated 16S rRNA and *rpoB* sequences of the *S. halichoeri* strains of different origin

Seventy-two (49 from canines, 23 from fur animals) of the 138 SHL isolates identified from the database report search were available for additional biochemical testing. The clinical isolates originated from different individuals, except in two cases, in which canine patients had two distinct isolates. The three *S. halichoeri* reference strains were also included. A positive catalase reaction was recorded for all isolates, including the reference strains, when bacterial mass was taken from a culture on a blood agar base carefully avoiding blood contamination. Cultures grown on a chromogenic agar (UriSelect4) gave the same result, but the reaction was slower and weaker. The majority (n = 69) of the characterized isolates and both human reference strains produced turquoise colonies on the chromogenic plate and their growth characteristics resembled those of enterococci, while four isolates (Strep B-11, Finnraccoon; Strep B-13, mink; Strep B-18, mink; and the seal CCUG 48324^T^) produced whitish colonies. These four isolates also shared a similar biochemical profile in API 20 Strep, all being esculin negative while the other isolates were esculin positive. Altogether, 17 different API profiles were observed, the most frequent one being 5563100 (30/75; 40%). This profile was observed in canine isolates only, and in both human CCUG reference strains. The second most common profile was 5163110, representing 18 (24%) isolates, of which 13 were of fur animal origin. These were followed by 5173510 (seven canine isolates; 9%). Table 3 summarizes the API profiles, and API profiles are also presented in Figs. 1 and 2 to observe the clustering of biochemical profiles according to PFGE and genetic clades, respectively. An APIWEB search resulted in nine low discrimination profiles, six unacceptable profiles, one doubtful profile and one good identification. The most common genus/species suggested by APIWEB were *Lactococcus lactis*, *Enterococcus faecium* and *durans*, *Aerococcus urinae*, *Streptococcus constellatus, Streptococcus porcinus*, and *Gemella haemolysans*. The profile 5163100 was detected only once and resulted a good identification of *L. lactis* with ID 91.2% and T 0.34.

**Table 3:** API 20 Strep profiles of canine and fur animal *Streptococcus halichoeri* isolates and reference strains

API 20 Strep code^a^	n (y)^b^	VP	HIP	ESC	PYRA	αGAL	βGUR	βGAL	PAL	LAP	ADH	RIB	ARA	MAN	SOR	LAC	TRE	INU	RAF	AMD	GLYG
5563100^c^	30	+	−	+	+	−	+	−	+	+	+	+	−	+	−	−	−	−	−	−	−
5163110	18 (13)	+	−	+	+	−	−	−	+	+	+	+	−	+	−	−	+	−	−	−	−
5173510	7	+	−	+	+	−	−	+	+	+	+	+	−	+	−	+	+	−	−	−	−
5173110	4	+	−	+	+	−	−	+	+	+	+	+	−	+	−	−	+	−	−	−	−
5173100	3 (3)	+	−	+	+	−	−	+	+	+	+	+	−	+	−	−	−	−	−	−	−
1163102	2 (2)	+	−	−	+	−	−	−	+	+	+	+	−	+	−	−	−	−	−	−	+
5561100	1	+	−	+	+	−	+	−	+	+	+	−	−	+	−	−	−	−	−	−	−
5561000	1	+	−	+	+	−	+	−	+	+	+	−	−	−	−	−	−	−	−	−	−
5173410	1	+	−	+	+	−	−	+	+	+	+	+	−	−	−	+	+	−	−	−	−
5163510	1 (1)	+	−	+	+	−	−	−	+	+	+	+	−	+	−	+	+	−	−	−	−
5163111	1	+	−	+	+	−	−	−	+	+	+	+	−	+	−	−	+	−	−	+	−
5163100	1	+	−	+	+	−	−	−	+	+	+	+	−	+	−	−	−	−	−	−	−
5163010	1 (1)	+	−	+	+	−	−	−	+	+	+	+	−	−	−	−	+	−	−	−	−
5163000	1 (1)	+	−	+	+	−	−	−	+	+	+	+	−	−	−	−	−	−	−	−	−
5161010	1 (1)	+	−	+	+	−	−	−	+	+	+	−	−	−	−	−	+	−	−	−	−
1163100^d^	1	+	−	−	+	−	−	−	+	+	+	+	−	+	−	−	−	−	−	−	−
1163103	1 (1)	+	−	−	+	−	−	−	+	+	+	+	−	+	−	−	−	−	−	+	+
Consensus profile^e^		+	−	V(+)	+	−	V (±)	V (−)	+	+	+	V (+)	−	V (+)	−	V (−)	V (±)	−	−	V (−)	V (−)

^a^API 20 Strep panel: VP, acetoin production (Voges Proskauer); HIP, hydrolysis of hippuric acid; ESC, β-glucosidase hydrolysis (esculin); PYRA, pyrrolidonyl arylamidase; αGAL, α-galactosidase; βGUR, β-glucuronidase; βGAL, β-galactosidase; PAL, alkaline phosphatase; LAP, leucine aminopeptidase; ADH, arginine dihydrolase; RIB, ribose; ARA, arabinose; MAN, mannitol; SOR, sorbitol; LAC, lactose; TRE, trehalose; INU, inulin; RAF, raffinose; AMD, amidon; GLYG, glycogen

^b^n (y), n indicates the total number of isolates with the respective API 20 Strep code and (y) number of isolates from fur animals with that code

^c^Includes the reference strains *S. halichoeri* “subsp. *hominis*” CCUG 61265 and CCUG 67100

^d^The reference strain *S. halichoeri* subsp. *halichoeri* CCUG 48324^T^

^e^Consensus profile shows reactions that are common to all isolates and biological variation in reactions between the isolates (n = 49 canine isolates, n = 23 fur animal isolates, and the reference strains (CCUG 48324^T^, CCUG 61265 and CCUG 67100); + positive, **−** negative; V(+) ≥ 90% of isolates positive; V(±) 40–60% of isolates positive; V(**−**) ≥ 80% of isolates negative

In the BLAST search for the trimmed 1400 bp 16S rRNA gene sequences of SHL isolates in the NCBI database, the *S. halichoeri* seal isolate CCUG 48324^T^ was the first hit in the identification, with the following identity percentages: 98.1% to 98.3% (53 isolates) and 99.5% (3 isolates). Of the three isolates with 99.5% identity, two were esculin-negative fur animal isolates and one was an esculin-positive isolate of canine origin. When compared with the human *S. halichoeri* “subsp. *hominis*” CCUG 61265 (or CCUG 67100) sequence, the sequence identities were as follows: 98.5% (n = 3; respective three isolates as above), 99.6–99.9% (n = 30) and 100% (n = 23). MALDI-TOF identified all isolates as *S. halichoeri* with high scores ranging from 2.030 to 2.380. Multi-drug resistance (MDR) to ERY, CLI and TET was recorded for 26/49 (53%) canine isolates, while 19/49 (39%) isolates were fully susceptible. The majority (70%, 16/23) of the fur animal isolates were fully susceptibility to the tested antimicrobials and none expressed simultaneous resistance to ERY and CLI, or inducible resistance to CLI (expressed as a D-shaped inhibition zone).

With PFGE, the 72 clinical isolates and three *S. halichoeri* reference strains were distributed in three major clusters (A–C) (Fig. 1). The main cluster A contained 38 (51%) isolates, and clusters B and C contained 23 (31%) and 14 (19%) isolates, respectively. The PFGE profile of the human strain CCUG 61265 was identical to 12 *S. halichoeri* isolates from dogs (cluster B). Of these, 11 canine and the human isolate also had identical API profiles (5563100). The other human type strain, CCUG 67100, clustered closely (96%) in PFGE with three canine isolates (P-317, P-628 and P-1072) also sharing an identical API profile (5563100). Two canine patients harboured isolates from two distinct clusters, B and C (P-791 and P-1013) as well as A and C (P-630 and P-629). No apparent temporal or spatial clustering was noted, except for the rare fur animal cases, where specimens were simultaneously taken from the same farm. Figure 1 summarizes the susceptibility results and biochemical profiles of the isolates together with the PFGE patterns.

Twenty-seven canine and 11 fur animal isolates, as well as three CCUG reference strains, were included in a phylogenetic tree based on concatenated sequences of 16S rRNA and *rpoB*. The phylogenetic tree is presented in Fig. 2 with the PFGE and API results. All *S. halichoeri* sequences were clearly monophyletic, and they formed two distinct clades that split further into subclusters. The first clade included the human isolates and had a higher support value. Within this cluster, the canine isolates shared common ancestry with the human isolates, while isolates from fur animals formed their own subcluster. The second clade, including the seal CCUG 48324^T^ strain, was in general less well supported and less structured, with sequences of seal, canine and fur animal origin mixed together, suggesting a recent, common source of origin for all these isolates.

## Discussion

Our study suggests that *S. halichoeri* is a relatively common finding in specimens from dogs and minks, but can also be found from blue foxes and Finnraccoons. We could not identify previous reports on the occurrence of *S. halichoeri* in canine infections. As we characterized a relatively high proportion of these isolates by biochemical and molecular methods, we can confirm the SHL species as *Streptococcus halichoeri*. MALDI-TOF identified the bacterium with high confidence, while 16S rRNA sequencing revealed 98–100% similarity with reference strains in the NCBI database. In general, our isolates shared a higher similarity with the human CCUG strains. In contrast to other streptococci, we observed that *S. halichoeri* isolates, including the CCUG reference strains, were catalase positive, even when cultivated on blood-free media, although the reaction was generally much slower and weaker compared to bacteria cultivated on blood-containing media. This is in contrast to earlier reports [1, 5], while otherwise the growth characteristics of our isolates were identical to previous descriptions. In one earlier study, however, positive catalase reactions for human and seal *S. halichoeri* isolates were observed, but were considered as false reactions, since this characteristic was detected only after growth on blood-containing media [5]. Further characterization of a selected set of *S. halichoeri* strains of this study by whole genome sequencing confirmed that they carried a catalase gene [15].

On the chromogenic agar we used in this study, the majority of isolates produced turquoise colonies resembling those of enterococci. The most frequent API 20 Strep profiles of our isolates were 5563100 (30 isolates, including two human CCUG strains) and 5163110 (18 isolates, of which 13 were from fur animals). Interestingly, all fur animal isolates were βGUR negative, while two-thirds of the canine isolates were βGUR positive. All isolates were esculin positive, except one canine and three fur animal isolates, which also formed their own subcluster in a phylogenetic tree (Fig. 2). As this bacterial species is currently lacking from the APIWEB database, its biochemical identification may be challenging. The positive catalase reaction may also hamper the identification. The findings of this study aid the identification of *S. halichoeri*, especially when MALDI-TOF identification is not available. They could be even utilized to update the current APIWEB database.

More than half of the canine *S. halichoeri* were resistant to ERY and CLI, and our data indicate that TET resistance is also frequent in canine *S. halichoeri*. A recent publication concerning infectious cellulitis in a human patient reported that a *S. halichoeri* “subsp. *hominis*” strain was resistant to ERY and CLI due to the presence of the *erm*B gene, and resistant to TET due to the *tet*O gene [7]. The *erm*B gene is frequent in streptococcal species such as *S. pneumoniae* [16]. Although resistance genes were not tested in our study, the phenotypic data indicate that the same mechanisms are frequent in canine isolates, while resistance is very uncommon in fur animal isolates.

The significance of *S. halichoeri* as a pathogen in fur animals and dogs is a matter of debate. Nordgren et al. [8] described *S. halichoeri* in FENP lesions of different fur animals together with *A. phocae*, while the former (and other streptococci) could not be isolated from healthy controls [17]. The direct culture technique, however, may not be sensitive enough to detect very small numbers of streptococci among normal microbiota. In diseased fur animals, *S. halichoeri* appears to have a predilection for mucocutaneous junctions, as the lesions were mostly situated around eyelids, nares, lips, paws or nailbeds. On the other hand, these are also predilection sites for lesions in FENP [8], and lesions may simply provide favourable conditions for the multiplication of commensals together with pathogen(s). The bacterium was only occasionally isolated from deeper infections such as in the lungs, uterus and other internal organs in fur animals. All *S. halichoeri* cases except one from fur animals were polymicrobial (mainly with *A. phocae*). The same applies to the canine findings. Canine skin and ear infections are often secondary due to underlying conditions that favour polymicrobial growth. Because *S. pseudintermedius* is considered to be of primary importance in these conditions, it is possible that non-haemolytic catalase-positive *S. halichoeri* colonies are simply ignored by clinical laboratories. In this study, *S. halichoeri* was observed in some canine abscesses and postsurgical infections, but never as the only species. If this were an actual pathogen of dogs, one could expect it to be more frequently observed in deeper specimens. The pathogenic potential of *S. halichoeri*, however, should not be neglected, as it has been associated with severe infections in other animal species. It has been isolated from post-mortem and other specimens in seals [1], a cerebral infection in an American black bear [18], pyogranulomatous pleuropneumonia in a European badger [2], and a post-mortem kidney specimen from a dead Steller sea lion (together with *S. phocae*) [3]. There are reports of severe human infections associated with *S. halichoeri*, such as sepsis and postoperative empyema [4, 5], underlining its pathogenic and zoonotic potential. According to a risk assessment report by Public Health England in 2013–2015 [19], this pathogen is unlikely to pose a significant zoonotic risk for the general population, but seal handlers and veterinarians may be at risk of exposure. However, considering the frequency of canine contacts in the general population, as well as the relatively common presence of this pathogen in canine skin/ear lesions, exposure of humans to this bacterium is more likely via canine contact than via seals or other marine sources. Moreover, the PFGE and sequence-based phylogenetic tree of our study demonstrated a genetic match of human *S. halichoeri* CCUG strains with several isolates of canine origin, indicating transmission of this bacterium between humans and dogs. We were able to find a case report of paronychia in a human who had had previous contact with a dog [6]. In order to assess the zoonotic risk and identify the possible source of the organism, physicians should also recognize dogs and probably fur animals as potential sources of *S. halichoeri* for humans. As this bacterium appears to exist in several animal species, we suggest that physicians ask patients about possible animal contacts prior to the onset of a *S. halichoeri* infection.

From an evolutionary perspective, *S. halichoeri* is an interesting species linking seals to fur animals, dogs and humans. *A. phocae*, which is considered to be of main importance in fur animal FENP, has also been associated with infections in grey seals and common seals (*Phoca vitulina*) [20]. The first occurrence of FENP in minks (pododermatitis at that time) in the 1970s and 1990s in the USA and Canada, respectively, was observed soon after the use of seal by-products in mink feed was initiated. As *S. halichoeri* is commonly associated with *A. phocae* in fur animal FENP, the use of seal by-products might explain the transfer of both bacterial species to the fur animal population. However, seal by-products have not been used in fur animal feed in Finland, although individual fur animals for breeding have been transported from countries known to use such feed. Another option is that *S. halichoeri* may be present in feed containing material from the fish industry, providing a continuous source of the bacterium to the fur animal population. The route of this bacterial species to the canine population is currently unknown, although direct contact with fur animals or fish-based feed—especially raw feed—might be an option, together with contacts with fur animals or their environment in fur farms.

It has been suggested that *S. halichoeri* should be renamed as *S. halichoeri* subsp. *halichoeri* and *S. halichoeri* “subsp. *hominis*” due to differences in biochemical patterns and 16S rRNA, *rpoB*, *sod*A, and *rec*N sequences between seal and human isolates [5]. In that study, each gene was compared individually. A phylogenetic tree based on concatenated 16S rRNA and *rpoB* genes in our study revealed two main clades, which further divided into two to three subclusters. The seal CCUG 48324^T^ isolate together with a couple of fur animal and canine isolates formed one of these subclusters. Likewise, they shared a similar biochemical pattern and clustered in PFGE. In general, the fur animal isolates tended to cluster into their own groups in both analyses, although some canine isolates were scattered among these. Canine isolates were observed in both main clades. The human CCUG strains were identical (CCUG 61265) or similar (CCUG 67100) in PFGE to the canine isolates. Fifteen canine and both human CCUG isolates formed an identical subcluster in the phylogenetic tree. However, regardless of the grouping and differences in biochemical patterns between the seal group and other groups, all the isolates were close to each other phylogenetically. Therefore, we consider that more isolates from different animal sources and more genetic data, preferably based on whole genome sequencing (WGS), are needed to assess whether division into two or more subspecies of *S. halichoeri* is reasonable.

## Conclusions

SHL isolates from dogs, minks, blue foxes and Finnraccoons were confirmed to be *S. halichoeri*. As this species rarely appeared as the only bacterial species from the sampled infections, the significance of this bacterium as a pathogen in these animals remains a matter of debate. As a wide set of isolates from different animals was characterised, the results of this study can be utilized for the identification of this bacterium. No apparent temporal or spatial clustering was detected, but isolates from different host species and individual animals were genetically very similar. Because many canine isolates were identical to the human reference strains, we postulate that dogs could be potential sources of *S. halichoeri* for humans. WGS sequencing of strains from different sources is needed in order to obtain a more accurate picture of the epidemiology and virulence of *S. halichoeri*, as well as to assess whether the division of this species into subspecies is necessary.

## Data Availability

The datasets used and/or analysed during the current study are available from the corresponding author on reasonable request.
